# Eukaryotic translation initiation factor 5A2 regulates the migration and invasion of hepatocellular carcinoma cells via pathways involving reactive oxygen species

**DOI:** 10.18632/oncotarget.8324

**Published:** 2016-03-23

**Authors:** Rong-Rong Liu, Ya-Su Lv, Yue-Xiao Tang, Yan-Fang Wang, Xiao-Ling Chen, Xiao-Xiao Zheng, Shang-Zhi Xie, Ying Cai, Jun Yu, Xian-Ning Zhang

**Affiliations:** ^1^ Department of Cell Biology and Medical Genetics, Research Center for Molecular Medicine, National Education Base for Basic Medical Sciences, Institute of Cell Biology, Zhejiang University School of Medicine, Hangzhou, 310058, China; ^2^ Department of Biological Chemistry, Zhejiang Chinese Medical University, Hangzhou, 310053, China; ^3^ Department of Hepatobiliary and Pancreatic Surgery, The First Affiliated Hospital, Zhejiang University School of Medicine, Key Laboratory of Multi-Organ Transplantation of Ministry of Public Health, Hangzhou, 310003, China

**Keywords:** eukaryotic translation initiation factor 5A2, reactive oxygen species, epithelial-mesenchymal transition, migration, hepatocellular carcinoma

## Abstract

Eukaryotic translation initiation factor 5A2 (*eIF5A2*) has been identified as a critical gene in tumor metastasis. Research has suggested that reactive oxygen species (ROS) serve as signaling molecules in cancer cell proliferation and migration. However, the mechanisms linking *eIF5A2* and ROS are not fully understood. Here, we investigated the effects of ROS on the eIF5A2-induced epithelial-mesenchymal transition (EMT) and migration in six hepatocellular carcinoma (HCC) cell lines. Western hybridization, siRNA transfection, transwell migration assays, wound-healing assays, and immunofluorescence analysis were used. The protein levels of eIF5A2 in tumor and adjacent tissue samples from 90 HCC patients with detailed clinical, pathological, and clinical follow-up data were evaluated. Overexpression of eIF5A2 was found in cancerous tissues compared with adjacent tissues. We found that eIF5A2 overexpression in HCC was associated with reduced overall survival. Knockdown of *eIF5A2* and intracellular reduction of ROS significantly suppressed the invasion and metastasis of HCC cells. Interestingly, N1-guanyl-1, 7-diaminoheptane (GC7) suppressed the intracellular ROS levels. After blocking the EMT, administration of GC7 or N-acetyl-L-cysteine did not reduce cell migration further. Based on the experimental data, we concluded that inhibition of eIF5A2 alters progression of the EMT to decrease the invasion and metastasis of HCC cells *via* ROS-related pathways.

## INTRODUCTION

Hepatocellular carcinoma (HCC) is one of the most common malignant tumors, with high morbidity and mortality rates worldwide, especially in the developing countries of East Asia [1–3]. It is responsible for > 600,000 deaths annually and ~750,000 new cases are diagnosed every year [1–3]. Metastasis, the phenomenon by which tumor cells break away from a primary site, circulate through the bloodstream, infiltrate vascular and lymphatic vessels, and settle and proliferate elsewhere in the body, is a complicated pathological process responsible for a poor prognosis [4]. Intervention in metastasis is a potential approach to treating cancer [5, 6]. Before migration, cells often exhibit a series of morphological changes, such as the epithelial-mesenchymal transition (EMT), which is recognized as a critical process that involves morphological changes and increases the invasiveness of cancer cells, cancer progression, and metastasis [7–10]. Cells that undergo the EMT often lose epithelial adhesion and cytoskeletal components, express mesenchymal components, and acquire a migratory phenotype [11]. Despite much research, the signaling mechanisms that trigger the EMT and migration are not fully understood.

Eukaryotic translation initiation factor 5A2 (eIF5A2), an isoform of eIF5A, is a well-conserved protein found in all organisms. *eIF5A2* on human chromosome 3q26.2 has been identified as a novel oncogene in cancer [12, 13]. eIF5A2 is the only cellular protein that contains the unusual amino-acid hypusine [N(ε)-(4-amino-2-hydroxybutyl)lysine]. Inhibition of eIF5A2 activity by N1-guanyl-1, 7-diaminoheptane (GC7), an inhibitor of deoxyhypusine synthase, has strong anti-tumor effects on human cancer cells [14]. For example, GC7 combination therapy enhances the therapeutic efficacy of doxorubicin in bladder cancer and estrogen-negative breast cancer cells by inhibiting eIF5A2 activation and preventing the EMT [15, 16]. Moreover, in many cancers, eIF5A2 plays a vital role in EMT progression by transcriptional inhibition of different downstream molecules [17, 18].

Excessive reactive oxygen species (ROS) can cause fatal lesions in cells under oxidative stress conditions, leading to many diseases including cancers [19]. The connection between ROS and cancer is complicated, because each type of ROS has a specific effect on cancer cells [20]. Increasing numbers of studies suggest a close correlation between ROS and cancer metastasis [21], i.e., ROS serve as signaling molecules in cancer cell proliferation and migration and can directly oxidize important cellular proteins [22].

In this study, we first analyzed the distribution of eIF5A2 expression in tissue microarrays to explore its relationship with prognosis. eIF5A2 was significantly overexpressed in human HCC tissue samples compared with adjacent tissues. Interestingly, GC7 reduced the intracellular ROS levels. Thus, we performed further experiments to investigate the roles of ROS in the eIF5A2-induced EMT and HCC invasion and metastasis. The results implied that inhibition of eIF5A2 reduces the invasion and metastasis of HCC cells *via* pathways involving ROS.

## RESULTS

### Inhibition of eIF5A2 reduced the invasion and migration of HCC cells *in vitro*

**Figure 1:**
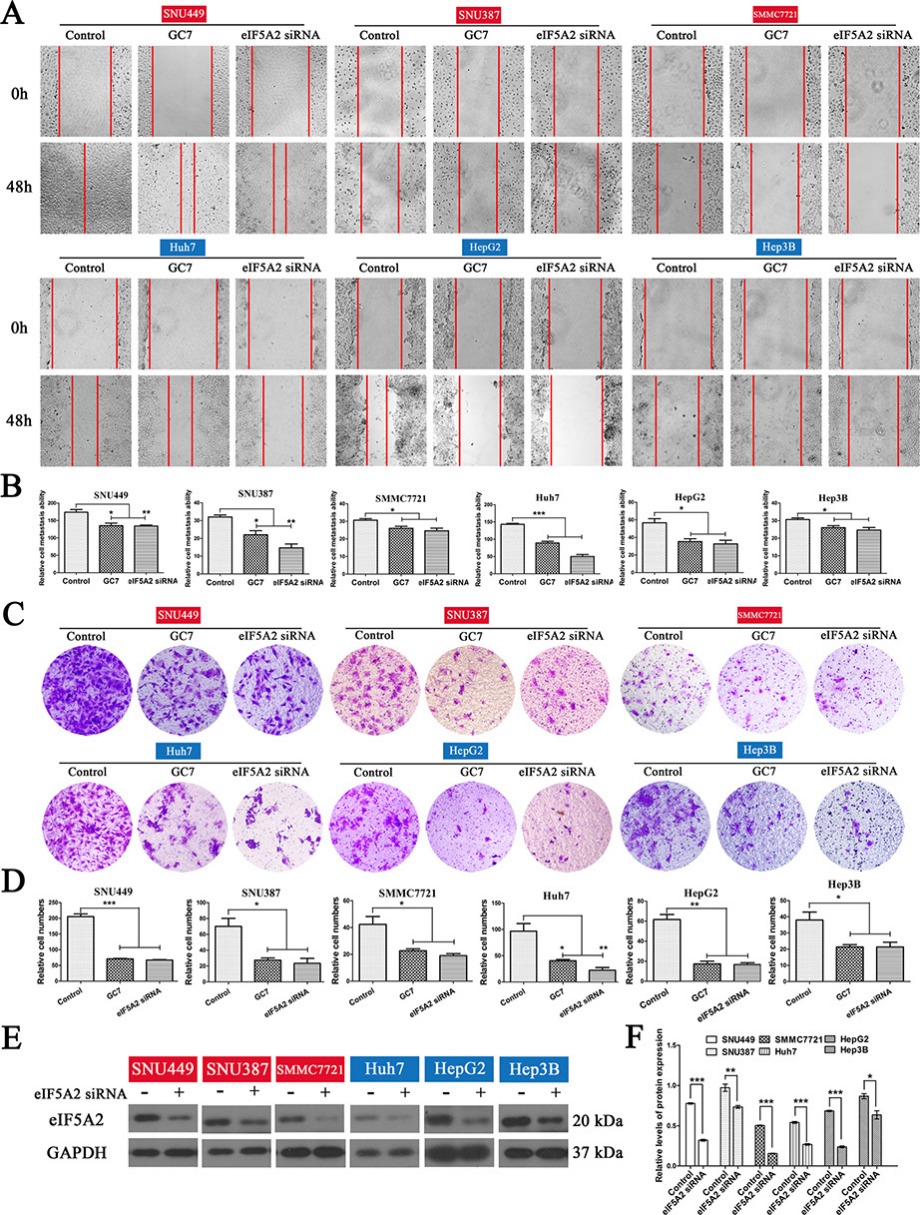
Effects of eIF5A2 on the invasion and metastasis of HCC cells (**A**) Representative phase-contrast images of wound closure of six HCC cell lines treated with GC7 (50 μM) or transfected with *eIF5A2* siRNA (100 nM) at 0 and 48 h after creating the wound (magnification 100 ×). (**B**) Bar graphs based on quantitative data from (A). Data are mean ± SEM. *P < 0.05, **P < 0.01, ***P < 0.001 *versus* control. (**C**) Representative photographs of invasion in the GC7 and *eIF5A2* siRNA groups compared with the control groups in transwell assays (magnification 100 ×). (**D**) Graphs based on quantitative data from (C). Data are mean ± SEM. *P < 0.05, **P < 0.01, ***P < 0.001 *versus* control. (**E**) Western hybridization confirming the effect of eIF5A2 siRNA transfection. (**F**) Bar graphs based on quantitative data from (E). Data are mean ± SEM. *P < 0.05, **P < 0.01, ***P < 0.001 *versus* control. Each experiment was repeated at least three times.

Wound-healing assays were performed to determine the association between eIF5A2 and the migratory capacity of HCC cells, while their invasiveness was tested by transwell assays. Based on previous reports and preliminary experiments, 50 μM GC7, which inhibits eIF5A2 activation, and 100 nM eIF5A2 siRNA, which silences eIF5A2 expression, were used. Wound-closure in the GC7 and *eIF5A2* siRNA groups was significantly slower than in the control groups. Particularly, changes in SUN449 cells demonstrated that suppression of *eIF5A2* reduced the migratory ability of HCC cells *in vitro* (Figure 1A, 1B). Interestingly, all six HCC cell lines showed lower invasiveness in the presence of GC7 or *eIF5A2* siRNA transfection (Figure 1C, 1D). To confirm the ability of *eIF5A2* siRNA transfection to knock down the expression of *eIF5A2*, western blotting was used to assess eIF5A2 protein in the six cell lines (Figure 1E, 1F).

### Inhibition of eIF5A2 reduced the expression levels of ROS-related genes

On the basis of the gene expression profiles of HCC cells under various conditions, we identified a possible correlation between the inhibition of eIF5A2 and gene expression changes. Interestingly, the expression of a large number of genes was affected in SUN449 cells treated with 50 μM GC7, and the results suggested that GC7 inhibits the expression levels of some genes (Supplementary Figure S1A, S1C), especially ROS-related genes (Figure S1B). Real-time PCR results also reflected the mRNA levels of ROS-related genes, such as *SOD1*, *CAT*, and *NOS3* (Figure S1D). To confirm the expression levels of ROS-related genes, western hybridization was used to assess the SOD1, SOD3, and NOS3 proteins in the six cell lines (Figure S1E). The SOD1, SOD3, and NOS3 expression in the six GC7-treated HCC cells was higher than in untreated HCC cells, especially SNU449 cells.

### The expression of eIF5A2 was higher in the nuclei of HCC cells

**Figure 2:**
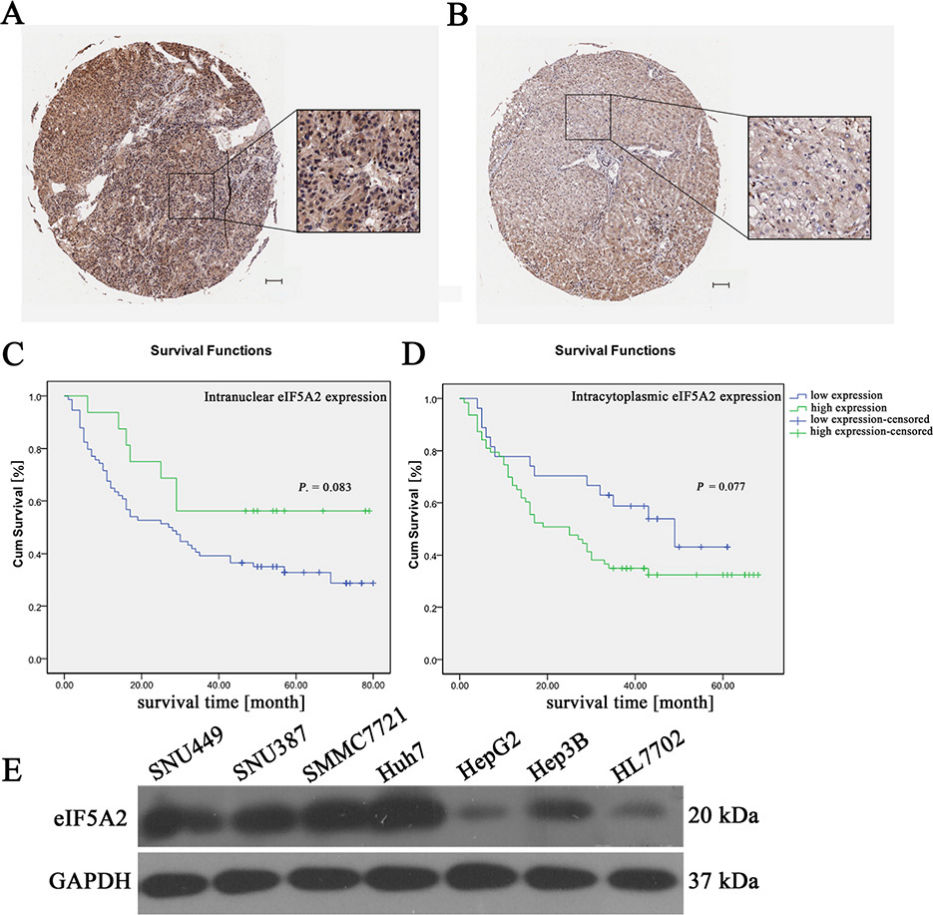
The relationship between eIF5A2 expression and overall survival (**A** and **B**) Representative immunohistochemical images showing the overexpression of eIF5A2 in HCC tissue (A) and its expression in adjacent tissue (B) (scale bars, 100 μm). (**C**) Kaplan-Meier curves for overall survival time in 90 HCC patients plotted according to their intranuclear expressions of eIF5A2; *P =* 0.083 by the log-rank test. (**D**) Kaplan-Meier curves for overall survival rates of 90 HCC patients based on the intracytoplasmic expression of eIF5A2; *P =* 0.077 by the log-rank test. (**E**) Western hybridization reflecting the expression of eIF5A2 in six HCC cell lines and normal human liver cells (HL7702).

To investigate the expression of eIF5A2 in HCC samples, an HCC tissue microarray containing 90 pairs of HCC specimens was analyzed. The results of nonparametric unpaired Wilcoxon tests showed that the expression of eIF5A2 in the nucleus of HCC samples was markedly higher than in adjacent tissues (*P =* 0.0001), but the eIF5A2 expression in the cytoplasm of HCC samples did not differ from that in adjacent tissues (*P =* 0.342) (Table 1). Immunohistochemical staining for the eIF5A2 protein in representative samples of HCC tissues and their paired adjacent tissues are shown in Figure 2A and 2B.

**Table 1:** Analysis of the differences in eIF5A2 expression between HCC and adjacent tissues

	Mean ± SD	*P*-value
Intranuclear		
eIF5A2 expression in HCC tissues	1.233 ± 2.249	0.0001
eIF5A2 expression in adjacent tissues	0.092 ± 0.547	
Intracytoplasmic		
eIF5A2 expression in HCC tissues	5.117 ± 2.958	0.342
eIF5A2 expression in adjacent tissues	4.622 ± 2.393	

### eIF5A2 overexpression was associated with poor overall survival and prognosis

Correlation analysis of clinicopathological characteristics and eIF5A2 expression showed that the expression of eIF5A2 was closely associated with node metastasis, distant metastasis, and tumor-node-metastasis staging (Table 2). Kaplan-Meier curves showed that eIF5A2 overexpression in HCC was associated with poor overall survival and prognosis. Intranuclear expression of eIF5A2 differed between HCC tissues and their paired adjacent tissues (*P =* 0.083) (Figure 2C). The intracytoplasmic expression of eIF5A2 showed a similar predisposition (*P =* 0.077) (Figure 2D). Compared to normal human liver cells (HL7702), we also found that the eIF5A2 expression was increased in all HCC cell lines except for HepG2 cells (Figure 2E).

**Table 2:** Correlation analysis of clinicopathological characteristics and eIF5A2 expression

Spearman's rank correlation coefficient		eIF5A2 expression in HCC tissues(cytoplasmic)	eIF5A2 expression in adjacent tissues(cytoplasmic)	eIF5A2 expression in HCC tissues(intranuclear)	eIF5A2 expression in adjacent tissues(intranuclear)	Gender	Age	Pathologial grade	Tumor size	T stage	Nodemetastasis	Distant metastasis	TNM staging
**eIF5A2 expression in HCC tissues (cytoplasmic)**	**Correlation coefficient**	1.000	.145	^.288**^	−.129	**−.008**	.122	^.210*^	.044	**.022**	−.024	.124	**.009**
	**Sig.2-tailed**	.	.174	**.006**	.235	.941	.256	**.047**	.683	.844	.832	.265	.934
	***N***	90	90	90	87	90	89	90	89	82	82	83	82
**eIF5A2 expression in adjacent tissues (cytoplasmic)**	**Correlation coefficient**	.145	1.000	−.068	**.005**	^−.249*^	.154	.129	.125	.193	.155	−.167	.172
	**Sig.2-tailed**	.174	.	.524	.960	**.018**	.149	.226	.243	.082	.163	.132	.121
	***N***	90	90	90	87	90	89	90	89	82	82	83	82
**eIF5A2 expression in HCC tissues (intranuclear)**	**Correlation coefficient**	^.288**^	−.068	1.000	.142	−.116	.122	.054	.058	−.082	.117	−.065	−.057
	**Sig.2-tailed**	**.006**	.524	.	.189	.277	.255	.611	.590	.461	.293	.557	.609
	***N***	90	90	90	87	90	89	90	89	82	82	83	82
**eIF5A2 expression in adjacent tissues (intranuclear)**	**Correlation coefficient**	−.129	**.005**	.142	1.000	−.060	.117	−.148	^−.214*^	−.102	**−.022**	**−.022**	−.101
	**Sig.2-tailed**	.235	.960	.189	.	.580	.284	.172	**.048**	.370	.845	.846	.375
	***N***	87	87	87	87	87	86	87	86	80	80	81	80
**Gender**	**Correlation coefficient**	**−.008**	^−.249*^	−.116	−.060	1.000	.086	−.100	−.154	−.079	−.037	**−.036**	−.077
	**Sig.2-tailed**	.941	**.018**	.277	.580	.	.424	.349	.148	.479	.745	.746	.493
	***N***	90	90	90	87	90	89	90	89	82	82	83	82
**Age**	**Correlation coefficient**	.122	.154	.122	.117	.086	1.00	**−.022**	−.188	−.074	^.298**^	**−.041**	**−.034**
	**Sig.2-tailed**	.256	.149	.255	.284	.424	.	.841	.079	.511	**.007**	.712	.762
	***N***	89	89	89	86	89	89	89	88	81	81	82	81
**Pathological grade**	**Correlation coefficient**	^.210*^	.129	.054	−.148	−.100	**−.022**	1.000	^.209*^	.209	**−.082**	.151	.197
	**Sig.2-tailed**	**.047**	.226	.611	.172	.349	.841	.	**.049**	.059	.463	.174	.077
	***N***	90	90	90	87	90	89	90	89	82	82	83	82
**Tumor size**	**Correlation coefficient**	**.044**	.125	.058	^−.214*^	−.154	−.188	^.209*^	1.000	^.684**^	.100	.101	^.684**^
	**Sig.2-tailed**	.683	.243	.590	**.048**	.148	.079	**.049**	.	**.000**	.374	.368	**.000**
	***N***	89	89	89	86	89	88	89	89	81	81	82	81
**T stage**	**Correlation coefficient**	**.022**	.193	−.082	−.102	−.079	−.074	.209	^.684**^	1.000	.094	.094	^.972**^
	**Sig.2-tailed**	.844	.082	.461	.370	.479	.511	.059	**.000**	.	.399	.399	**.000**
	***N***	82	82	82	80	82	81	82	81	82	82	82	82
**Node Metastasis**	**Correlation coefficient**	**−.024**	.155	.117	**−.022**	**−.037**	^.298**^	−.082	.100	.094	1.000	**−.012**	.205
	**Sig.2-tailed**	.832	.163	.293	.845	.745	**.007**	.463	.374	.399	.	.912	.064
	***N***	82	82	82	80	82	81	82	81	82	82	82	82
**Distant metastasis**	**Correlation coefficient**	.124	−.167	−.065	**−.022**	**−.036**	**−.041**	.151	.101	.094	−.012	1.000	.205
	**Sig.2-tailed**	.265	.132	.557	.846	.746	.712	.174	.368	.399	.912	.	.064
	***N***	83	83	83	81	83	82	83	82	82	82	83	82
**TNM staging**	**Correlation coefficient**	**.009**	.172	−.057	−.101	−.077	**−.034**	.197	^.684**^	^.972**^	.205	.205	1.000
	**Sig.2-tailed**	.934	.121	.609	.375	.493	.762	.077	**.000**	**.000**	.064	.064	.
	***N***	82	82	82	80	82	81	82	81	82	82	82	82

### GC7 reduced intracellular ROS levels

**Figure 3:**
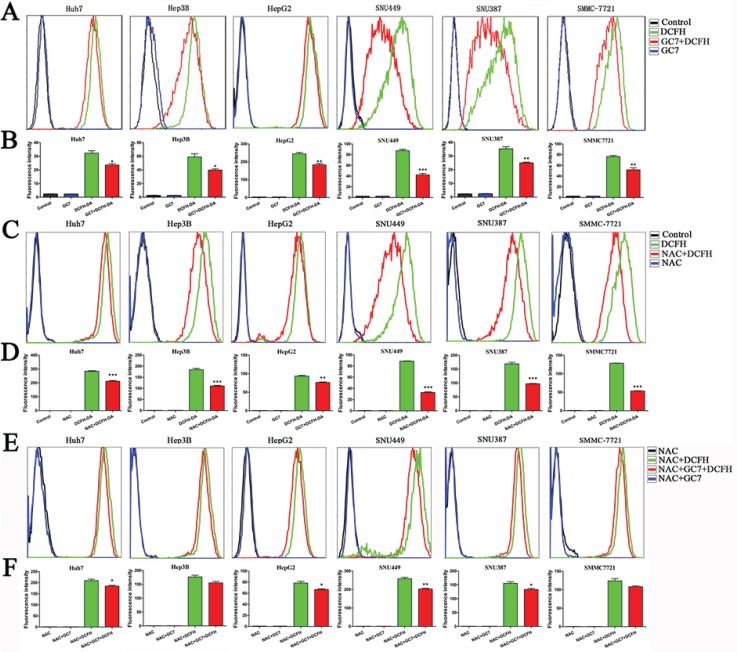
The changes of ROS levels under different conditions (**A**) ROS levels quantified by flow cytometry in the presence or absence of 30 μM GC7, compared with the control group. The fluorescence intensity was used to reflect the ROS levels with DCFH-DA (20 μM), which is a common probe. (**B**) Bar graphs showing the data based on (A). Data are mean ± SEM. *P < 0.05, **P < 0.01, ***P < 0.001 *versus* control. (**C**) The fluorescence intensity changes showed that NAC eliminated cellular ROS compared with the control group probed by DCFH-DA. (**D**) Bar graphs showing the data according to (C). Data are mean ± SEM. *P < 0.05, **P < 0.01,***P < 0.001 *versus* control. (**E**) The changes of ROS levels in six HCC cell lines with 50 μM NAC in the presence or absence of 30 μM GC7, compared with the control group. The fluorescence intensity was used to reflect the ROS levels. (**F**) Bar graphs showing the data based on (E). Data are mean ± SEM. *P < 0.05, **P < 0.01,***P < 0.001 *versus* control. Each experiment was repeated at least three times.

To evaluate the changes in cellular ROS levels after GC7 treatment (50 μM) for 24 h, dichlorodihydro-fluorescein diacetate (DCFH-DA) was used as a probe. The mean fluorescence intensity from flow cytometry revealed that GC7 significantly reduced the intercellular levels of ROS (Figure 3A, 3B). N-acetylcysteine (NAC), a ROS inhibitor, eliminated cellular ROS (Figure 3C, 3D). GC7 did not reduce the intercellular levels of ROS further in the six HCC cell lines treated with 50 μM NAC (Figure 3E, 3F).

### Inhibition of ROS suppressed invasion and metastasis in HCC cells

**Figure 4:**
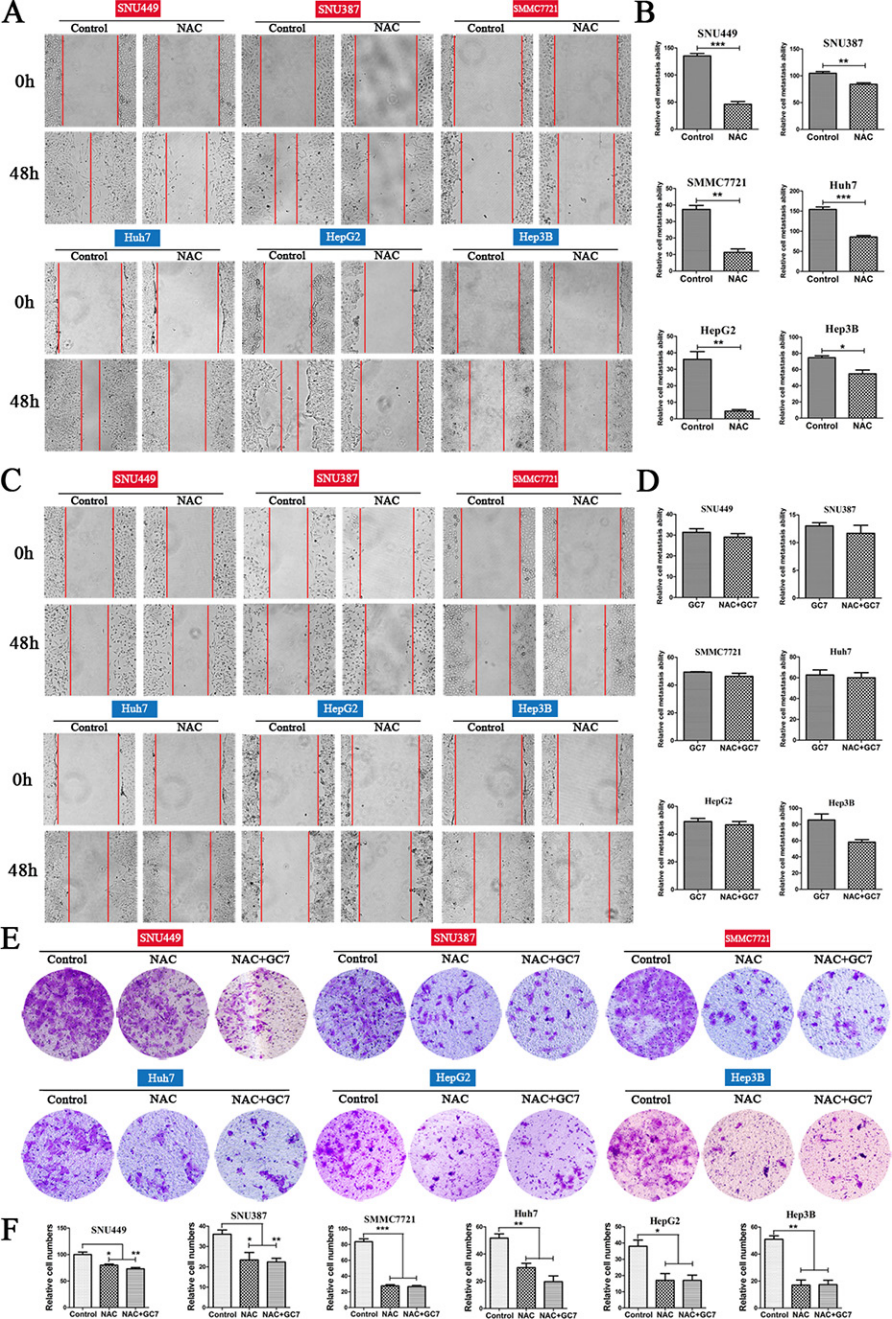
Effects of NAC and NAC plus GC7 on the invasion and metastasis of HCC cells (**A**) Wound-closure in the presence or absence of NAC (representative phase-contrast images at 0 and 48 h after creating the wound; magnification 100 ×). (**B**) Bar graphs based on quantitative data from (A). Data are mean ± SEM. *P < 0.05, **P < 0.01,***P < 0.001 *versus* control. (**C**) Wound-closure of cells cultured with NAC (50 μM) in the presence or absence of GC7 (magnification 100 ×). (**D**) Graphs based on quantitative data from (C). Data are mean ± SEM. (**E**) Representative photographs of transwell experiments showing that NAC suppressed cell invasion. (**F**) Bar graphs based on quantitative data from (E). Data are mean ± SEM. *P < 0.05, **P < 0.01,***P < 0.001 *versus* control. Each experiment was repeated at least three times.

To investigate whether the inhibition of ROS had any effect on the invasion and metastasis of HCC cells, the cell lines were cultured with NAC, which suppresses intracellular ROS. NAC significantly suppressed metastasis in liver cancer cells (Figure 4A, 4B). Notably, the results of transwell assays also showed that the NAC group had significantly lower invasiveness than the control group (Figure 4E, 4F).

### GC7 did not further suppress invasion and migration in HCC cells after NAC treatment

Interestingly, there was no significant difference in the metastatic activity of cells treated with NAC plus GC7 and those with NAC alone. This indicated that GC7 did not further suppress the invasion and migration in HCC cells treated with NAC (Figure 4C, 4D). Similarly, the results of transwell invasion assays showed that GC7 did not inhibit invasion when cells were treated with NAC (Figure 4E, 4F).

### Inhibition of eIF5A2 and ROS level changes reversed the EMT in HCC cells

**Figure 5:**
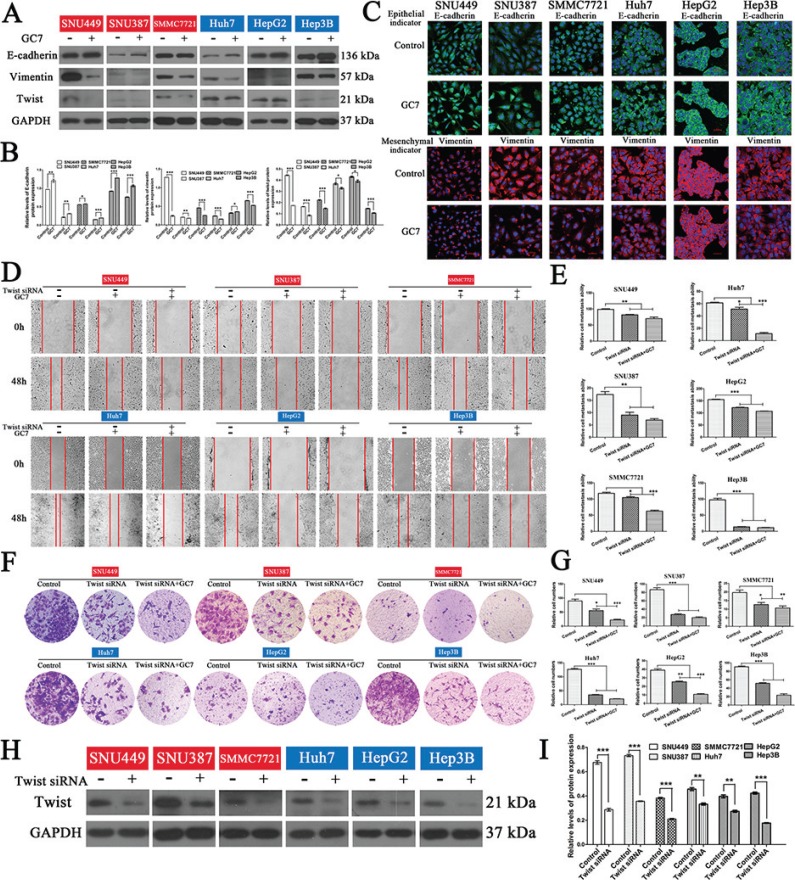
GC7 altered EMT progress and did not inhibit the invasion and migration of HCC cells after blocking the EMT (**A**) Expression of E-cadherin, vimentin, and twist in cells were cultured in the presence or absence of GC7 (50 μM) for 24 h. GAPDH served as a loading control. (**B**) Bar graphs based on quantitative data from (A). Data are mean ± SEM. *P < 0.05, **P < 0.01,***P < 0.001 *versus* control. (**C**) Immunofluorescence staining for E-cadherin (green), an epithelial marker, and vimentin (red), a mesenchymal marker, in six HCC cell lines cultured in the presence or absence of GC7 for 24 h. Nuclei were counterstained with DAPI. Scale bars, 50 μm. (**D**) Representative photographs of wound-healing assays at 0 and 48 h after creating a wound (magnification 100 ×). Cells were divided into three groups: control, GC7, and GC7 plus *twist* siRNA transfection. (**E**) Bar graphs based on quantitative data from (D). Data are mean ± SEM. *P < 0.05, **P < 0.01,***P < 0.001 *versus* control. (**F**) Representative photographs of crystal violet-stained cells after *twist* siRNA transfection in the presence or absence of GC7 for 48 h in transwell experiments (magnification 100 ×). (**G**) Bar graphs based on quantitative data from (F). Data are mean ± SEM. *P < 0.05, **P < 0.01,***P < 0.001 *versus* control. (**H**) Western hybridization confirmed the effect of *twist* siRNA transfection. (**I**) Bar graphs based on quantitative data from (H). Data are mean ± SEM. *P < 0.05, **P < 0.01,***P < 0.001 *versus* control. Each experiment was repeated at least three times.

**Figure 6:**
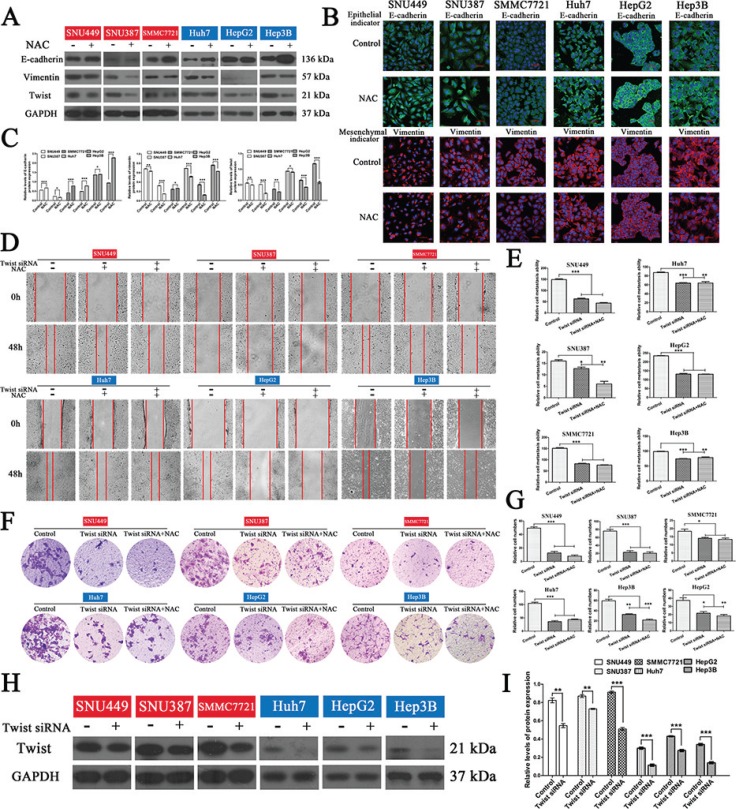
NAC reversed the EMT in HCC cells and the inhibitory effects on invasion and migration of HCC cells were reduced after blocking the EMT (**A**) Western blotting and (**B**) inverted confocal microscopic images (scale bars, 50 μm) showing the expression of E-cadherin (epithelial marker: green) and vimentin (mesenchymal marker: red) in HCC cells. Nuclei were counterstained with DAPI. (**C**) Bar graphs based on quantitative data from (A). Data are mean ± SEM. *P < 0.05, **P < 0.01,***P < 0.001 *versus* control. (**D**) Wound-closure control, NAC, and NAC plus *twist* siRNA transfection groups. Representative photographs at 0 and 48 h after creating a wound (magnification 100 ×). (**E**) Bar graphs based on quantitative data from (D). Data are mean ± SEM. *P < 0.05, **P < 0.01, ***P < 0.001 *versus* control. (**F**) Transwell chambers were used to determine the invasion of cells after *twist* siRNA transfection in the presence or absence of NAC for 48 h (representative photographs, magnification 100 ×). (**G**) Bar graphs based on quantitative data from (F). Data are mean ± SEM. *P < 0.05, **P < 0.01,***P < 0.001 *versus* control. (**H**) Western hybridization confirmed the effect of *twist* siRNA transfection. (**I**) Bar graphs based on quantitative data from (H). Data are mean ± SEM. *P < 0.05, **P < 0.01,***P < 0.001 *versus* control. Each experiment was repeated at least three times.

Since eIF5A2 triggers progression of the EMT [15], it is likely that inhibition of eIF5A2 reverses the EMT in HCC cells by affecting the expression of EMT markers. To test this hypothesis, the expression of epithelial and mesenchymal markers in liver cancer cells treated with GC7 or NAC for 24 h was assessed. We found that the administration of GC7 or NAC significantly enhanced the expression of E-cadherin (an epithelial marker), and decreased the expression of vimentin (a mesenchymal marker) in HCC cells, indicating that GC7 and NAC reverse progression of the EMT (Figure 5A, 5B, 6A, 6C). Moreover, the results of immunofluorescent staining were consistent with those of western blotting (Figure 5C, 6C).

### The effects of GC7 and NAC on invasion and migration in HCC cells were partially suppressed after blocking the EMT

In order to determine whether the effects of GC7 or NAC on invasion and metastasis act *via* the EMT in HCC cells, small interfering RNA (siRNA) experiments were performed to knock down the expression of twist to block the EMT in HCC cells. So, cells transfected with *twist* siRNA were incubated with NAC or GC7 for 24 h. Interestingly, the wound-healing and transwell assays revealed that the invasion and metastasis of cells treated with GC7 plus *twist* siRNA (Figure 5D, 5G) or NAC plus *twist* siRNA (Figure 6D, 6G) did not differ significantly from *twist* siRNA-transfected cells. To confirm the effect of *twist* siRNA transfection, western hybridization was performed to detect the twist expression in the six cell lines (Figure 5H, 5I, 6H, 6I). Taken together, these data demonstrated that the effects of GC7 and NAC on the invasion and migration of HCC cells were partly suppressed after blocking the EMT.

## DISCUSSION

Recent studies have reported that *eIF5A2* is aberrantly expressed in several types of tumor cells, including rectal carcinoma, gastric cancer, ovarian cancer, bladder cancer, and colon cancer [18, 23–25]. Thus, it is believed that *eIF5A2* is a vital oncogene in carcinogenesis. We detected the expression profiles of eIF5A2 in HCC tissues to explore the correlation between eIF5A2 and tumor prognosis. Compared with adjacent tissues, the expression of eIF5A2 in the nucleus of human HCC samples was markedly higher (*P =* 0.0001). In contrast, the intracytoplasmic expression of eIF5A2 in HCC tissues was somewhat lower than in adjacent tissues (*P =* 0.342). Kaplan-Meier curves clearly showed that eIF5A2 overexpression was associated with poor overall survival and the prognosis of HCC. The *P* value as analyzed by the log-rank test, which reflected the relationship between the intranuclear expression of eIF5A2 and overall survival in 90 HCC patients, was 0.083. In comparison, the *P* value of the intracytoplasmic expression of eIF5A2 was 0.077. These results suggested that eIF5A2 overexpression might play a role in the prognosis of HCC, although the *P*-values of the cytoplasmic expression of eIF5A2 were not less than 0.05, which might be explained by the small number of tissue samples investigated.

The EMT plays a vital role in cancer invasion and metastasis [26]. Moreover, previous reports have demonstrated that eIF5A2 is also associated with EMT progression in many cancers including colorectal carcinoma and HCC [18, 27]. However, the underlying mechanisms are not fully understood. Previous studies have shown that eIF5A2 eliminates the augmentation of carcinoma cell migration, invasion, and the EMT through down-regulating MTA1 (metastasis-associated 1) [18]. Interestingly, here we found relevant relationships between eIF5A2 and ROS gene expression as GC7 significantly reduced the intracellular levels of ROS. It is noteworthy that ours is the first to report this. To identify which genes might be affected by GC7, we treated cells with GC7 and used a microarray to identify genes showing differences in expression. The heat-map indicated that the mRNA levels of the ROS-related genes *SOD1*, *SOD3*, and *NOS3* were affected. Superoxide dismutases (SODs) are enzymes that catalyze the removal of superoxide free-radicals [28]. The expression of SOD1 and SOD3 increased, and this may contribute to the elimination of intracellular ROS levels. These clues hinted at the effects of ROS on the eIF5A2-mediated EMT.

Previous studies have reported that the ROS-regulated genes relevant to the EMT, metastasis, and invasion include E-cadherin, integrin, and matrix metalloproteinases [29–31]. As signaling messengers, ROS are able to oxidize critical target molecules such as protein kinase C and protein tyrosine phosphatases [32]. In this research, we also found that ROS induced the EMT in HCC cells. Inhibition of ROS by NAC resulted in E-cadherin up-regulation and vimentin down-regulation. It is interesting that ROS suppression by NAC also inhibited the EMT progression induced by eIF5A2, which confirmed the effects of ROS on the process of eIF5A2-mediated EMT. Furthermore, our *in vitro* studies showed that ROS was responsible for the invasion and metastasis of HCC cells induced by eIF5A2, as the administration of GC7 did not attenuate the invasion and metastasis of HCC cells further after reduction of the ROS levels by NAC. The fact that the inhibitory effects of GC7 and NAC on invasion and migration were attenuated after blocking the EMT showed that both GC7 and NAC act *via* the EMT to suppress the invasion and migration of HCC cells.

In conclusion, our results indicated that inhibition of eIF5A2 reverses the EMT to reduce invasion and metastasis *via* ROS-related pathways. ROS might serve as potential targets in eIF5A2-induced HCC invasion and metastasis [33]. Therefore, further studies on how eIF5A2 changes ROS levels to increase cell migration are required.

## MATERIALS AND METHODS

### Cell lines and cell cultures

The six human HCC cell lines SNU387, SNU449, SMMC7721, Huh7, Hep3B, and HepG2 were obtained from the American Type Culture Collection (ATCC, Manassas, VA, USA). Huh7 and HepG2 cells were cultured in DMEM (Gibco, Carlsbad, CA, USA) supplemented with 10% fetal bovine serum (FBS) (Gibco). Hep3B cells were cultured in MEM (Gibco) with 10% FBS. SNU387, SNU449 and SMMC7721 cells were cultured in RPMI-1640 medium (Gibco) containing 10% FBS. All cells were maintained at 37°C in a humidified atmosphere containing 5% CO_2_ in air.

### Chemicals and antibodies

NAC and DCFH-DA were from Sigma-Aldrich (St. Louis, MO, USA). GC7 was from Calbiochem (San Diego, CA, USA). The *eIF5A2* siRNA, *Twist 1* siRNA, *Twist 2* siRNA, and the negative control siRNA were from Santa Cruz Biotechnology (Santa Cruz, CA, USA). The X-tremeGENE siRNA transfection reagent was from Roche (Roche Applied Science, Penzberg, Germany). Trizol reagent was from Invitrogen (Carlsbad, CA, USA). Monoclonal rabbit anti-human antibodies against E-cadherin and vimentin, and monoclonal mouse anti-human antibodies against Twist, eIF5A2, and GAPDH were from Santa Cruz Biotechnology. Secondary anti-rabbit HRP-conjugated antibodies and secondary anti-mouse HRP-conjugated antibodies were from Bioworld (Louis Park, MN, USA).

### Intracellular ROS measurement

DCFH-DA is a widely-used probe for cellular ROS. Cells (2 × 10^5^/well) in a 6-well plate were incubated with GC7 (50 μM) or NAC (50 μM) for 24 h. After incubation in serum-free medium containing 20 μM DCFH-DA for 20 min, cells in each well were washed three times with phosphate buffer solution (PBS), digested by pancreatin, and immediately subjected to ROS measurement by flow cytometry (Cytomics FC 500 MCL, Beckman Coulter, USA). The wavelength was 488 nm for excitation and 525 nm for emission.

### Western hybridization

Cells were cultured overnight at 5 × 10^4^ cells per well in six-well tissue culture plates. After adherence, the cells were treated with GC7 (50 μM) or NAC (50 μM) for 24 h. To assess the protein levels of E-cadherin, vimentin, and twist, cells were washed twice in ice-cold PBS and lysed in 100 μL cell lysis buffer (Cell Signaling, Danvers, MA, USA) with protease inhibitors (Sigma-Aldrich). After centrifugation, the supernatants were collected and protein concentrations were measured using a BCA protein kit (Thermo Fisher, Rockford, IL, USA). Cell lysates (50 μg/lane) were separated on 10% SDS-PAGE and proteins were transferred to polyvinylidene difluoride (PVDF) membranes (Millipore, Billerica, MA, USA). The membranes were then blocked with Tris-buffered saline (TBS) containing 0.1% Tween 20 (TBS/T) and 5% bovine serum albumin (BSA). The PVDF membranes were then incubated with rabbit monoclonal anti-E-cadherin, anti-vimentin (1:1 000), and mouse monoclonal anti-twist, anti-GAPDH, and anti-eIF5A2 (1:1 000) antibodies overnight at 4°C, washed three times with TBS/T, and then incubated with the appropriate HRP-conjugated secondary antibodies (1:1 000) for 2 h at room temperature. The protein bands were detected by chemiluminescence (GE Healthcare, Piscataway, NJ, USA) and visualized by autoradiography on X-ray films (Kodak, Rochester, NY, USA). The protein levels were normalized to that of GAPDH.

### Small interfering RNAs

Cells were transfected with *eIF5A2* siRNA, *Twist* 1 siRNA, *Twist* 2 siRNA, or negative control siRNA (100 nM) using the X-tremeGENE siRNA transfection reagent (Roche), according to the manufacturer's instructions. The transfection medium (Opti-MEM; Gibco) was replaced with culture medium 8 h after transfection. All subsequent experiments were performed 24 h after transfection and repeated in triplicate.

### Transwell-Matrigel invasion assay

After transfection with *eIF5A2* siRNA, *Twist* 1 siRNA, or *Twist* 2 siRNA (100 nM) for 8 h or treatment with GC7 or NAC for 24 h, cells were seeded at 2 × 10^5^ cells/well in the upper chamber of 8-μm pore-size Transwell 24-insert plates (Corning, NY, USA) with appropriate medium without FBS. The upper chambers had been coated previously with 30 μg Matrigel (BD Biosciences, San Jose, CA, USA) and the lower chamber contained the appropriate medium with 10% FBS. After 48 h, cells on the upper side of the filters were cleansed with cotton swabs, and those on the bottom of the inserts were fixed in 4% paraformaldehyde for 30 min and stained with 0.05% crystal violet. Cells that had invaded to the lower surface were counted in at least 10 fields using an inverted phase-contrast microscope (Olympus; magnification, 20 ×) and photographed. Each experiment was repeated at least three times.

### Wound-healing assay

Cells were seeded into six-well plates at 2 × 10^5^ cells/well and cultured for 16–24 h until cells reached ~90% confluence. After transfection with *eIF5A2*, *Twist* 1, or *Twist* 2 siRNA (10 μmol/mL), or treatment with GC7 or NAC for 24 h, a scratch wound was made with a sterile pipette tip in the middle of the confluent monolayer, and the medium was changed to the appropriate medium without FBS after the detached cells were washed away. Photographs were taken using an inverted phase-contrast microscope (Olympus; magnification, 40 ×) at 0, 24, and 48 h. The closing of the scratch wound was measured and wound closure was expressed as a percentage compared with the size of the initial opening caused by scratching. Each experiment was repeated at least three times.

### Immunofluorescence confocal microscopy

Cells (5 × 10^4^ cells/mL) were maintained in 15-mm round-bottomed culture dishes (Nest Biotechnology, Ltd, China) for 24 h under various conditions, fixed with 4% paraformaldehyde for 30 min, permeabilized for 10 min at room temperature with 0.5% Triton X-100 in PBS, and blocked with 1% BSA for 1 h. Cells were incubated overnight at 4°C with primary antibodies recognizing E-cadherin and vimentin (1:200) in 5% FBS. The cells were then incubated with Alexa-Fluor 488-conjugated anti-rabbit (1:200) and Alexa-Fluor 550-conjugated anti-mouse (1:200) antibodies for 2 h at room temperature, washed three times with PBS, and mounted with 4′, 6-diamidino-2-phenylindole (DAPI) (Sigma). Fluorescence was visualized with an inverted confocal microscope (Nikon A1R; magnification, 40 ×). Images were captured using a Nikon digital camera and analyzed by NIS-Elements Viewer software. Each experiment was repeated at least three times.

### Statistical analysis

Data are presented as mean ± standard error of the mean (SEM). In Table 1, data are presented as mean ± standard deviation (SD). For statistical analysis, unpaired *t*-tests were applied. A *P* value < 0.05 was considered statistically significant. All experiments were performed independently in triplicate.

## SUPPLEMENTARY MATERIALS FIGURES


