# Supplementary material for: Eukaryotic translation initiation factor 5A2 regulates the migration and invasion of hepatocellular carcinoma cells via pathways involving reactive oxygen species
Source: Oncotarget. 2016 Mar 23;7(17):24348–60. doi: 10.18632/oncotarget.8324 (PMC5029706; doi:10.18632/oncotarget.8324)
Supplement: Supplementary file 1 [file oncotarget-07-24348-s001.pdf]

Eukaryotic translation initiation factor 5A2 regulates the migration and invasion of hepatocellular carcinoma cells *via* pathways involving reactive oxygen species

Supplementary Materials

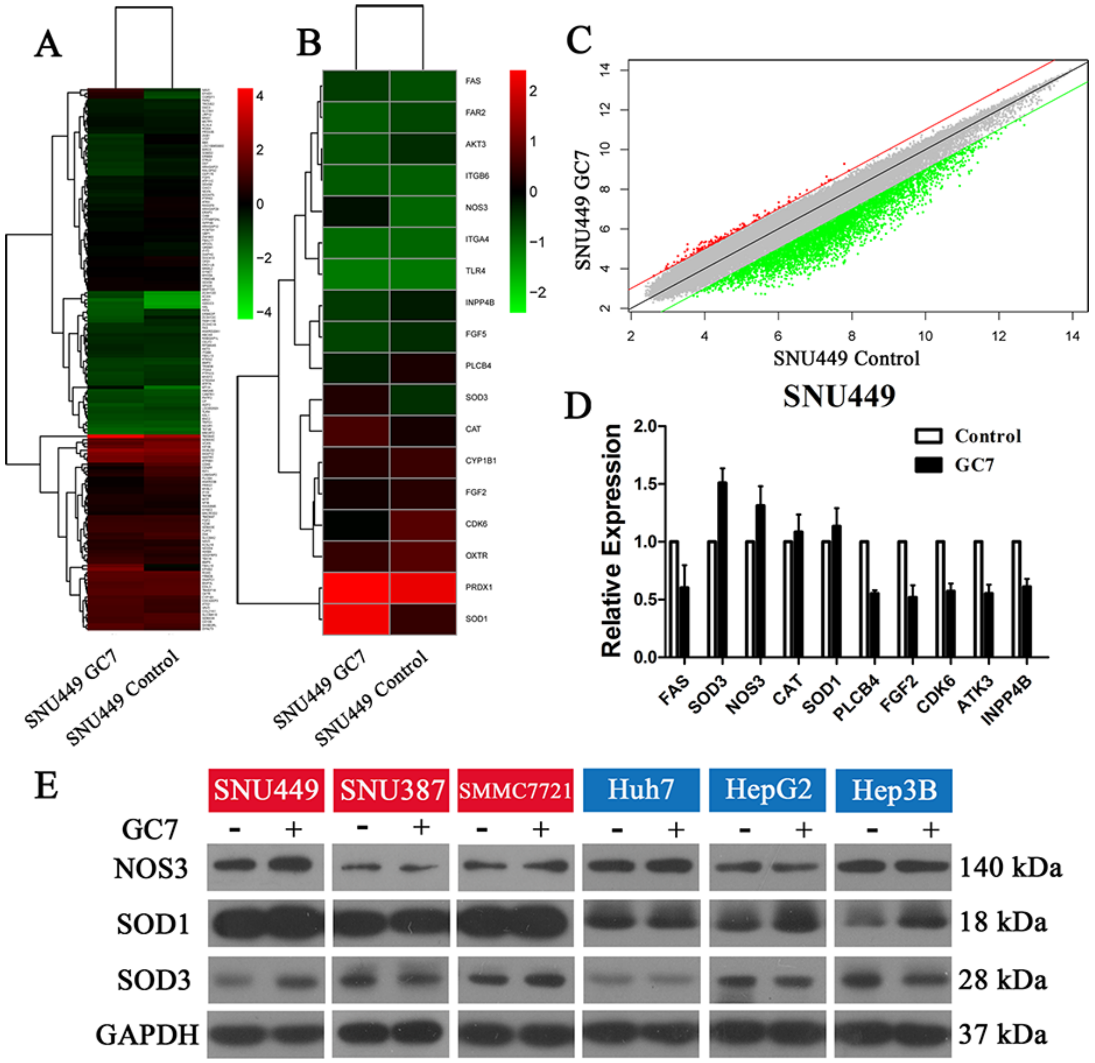

Supplementary Figure S1: Gene-chip screening revealed many differences in the expression of genes in HCC cells. (**A**) Microarray heat-map showed the differences in the expression profiles of SNU449 cells (fold change ≥ 2) treated with GC7 (red, up-regulation; green, down-regulation). (**B**) Microarray heat-map of ROS-related gene expression changes in SNU449 cells treated with GC7 (red, up-regulation; green, down-regulation) (fold change ≥ 2). (**C**) Scatterplot of expression changes in SNU449 cells treated with GC7 (red, up-regulation, fold change ≥ 2; gray, no significant change, fold change < 2; green, down-regulation, fold change ≥ 2) (**D**) Real-time PCR was performed to confirm the *mRNA* differences based on microarray heat-maps at the transcription level. .Data are mean ± SEM. (**E**) Western blotting of ROS gene expression: *SOD1*, *SOD3*, and *NOS3*. Each experiment was repeated at least three times.

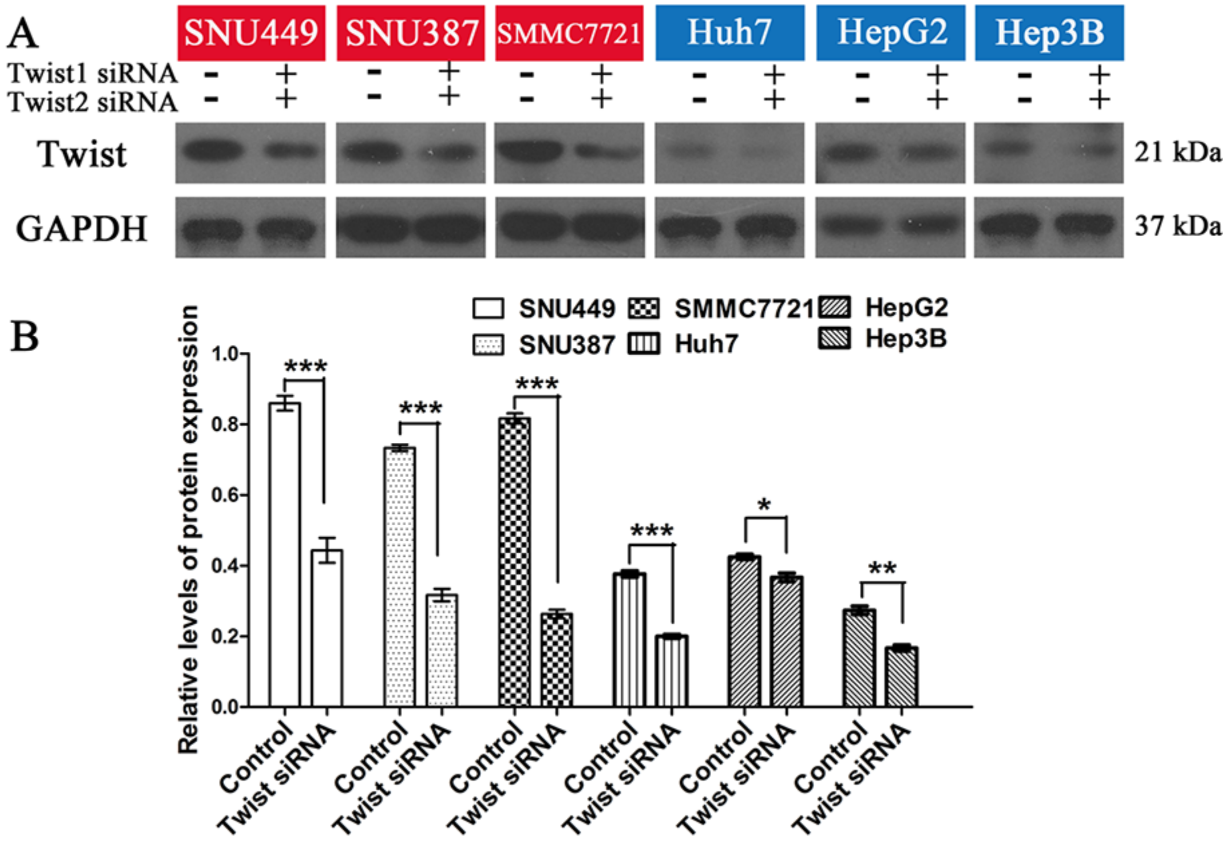

Supplementary Figure S2: (**A**) Western hybridization confirmed the success of ***twist1*/2** siRNA transfection. (**B**) Bar graphs based on quantitative data from (A). Data are mean ± SEM. *$P < 0.05$, **$P < 0.01$,***$P < 0.001$ *versus* control. Each experiment was repeated at least three times.
